# Suburothelial Bladder Contraction Detection with Implanted Pressure Sensor

**DOI:** 10.1371/journal.pone.0168375

**Published:** 2017-01-06

**Authors:** Steve J. A. Majerus, Paul C. Fletter, Elizabeth K. Ferry, Hui Zhu, Kenneth J. Gustafson, Margot S. Damaser

**Affiliations:** 1 Advanced Pltatform Technology Center, Louis Stokes Veterans Affairs Medical Center, Cleveland, OH, United States of America; 2 Department of Electrical Engineering and Computer Sciences, Case Western Reserve University, Cleveland, OH, United States of America; 3 Department of Biomedical Engineering, Cleveland Clinic, Cleveland, OH, United States of America; 4 Division of Urology, Louis Stokes Veterans Affairs Medical Center, Cleveland, OH, United States of America; 5 Urology Institute, University Hospitals, Case Medical Center, Cleveland, OH, United States of America; 6 Glickman Urological and Kidney Institute, Cleveland Clinic, Cleveland OH, United States of America; 7 Department of Biomedical Engineering, Case Western Reserve University, Cleveland, OH, United States of America; 8 Functional Electrical Stimulation Center, Louis Stokes Veterans Affairs Medical Center, Cleveland, OH, United States of America; University of Houston, UNITED STATES

## Abstract

**Aims:**

Managing bladder pressure in patients with neurogenic bladders is needed to improve rehabilitation options, avoid upper tract damage, incontinence, and their associated co-morbidities and mortality. Current methods of determining bladder contractions are not amenable to chronic or ambulatory settings. In this study we evaluated detection of bladder contractions using a novel piezoelectric catheter-free pressure sensor placed in a suburothelial bladder location in animals.

**Methods:**

Wired prototypes of the pressure monitor were implanted into 2 nonsurvival (feline and canine) and one 13-day survival (canine) animal. Vesical pressures were obtained from the device in both suburothelial and intraluminal locations and simultaneously from a pressure sensing catheter in the bladder. Intravesical pressure was monitored in the survival animal over 10 days from the suburothelial location and necropsy was performed to assess migration and erosion.

**Results:**

In the nonsurvival animals, the average correlation between device and reference catheter data was high during both electrically stimulated bladder contractions and manual compressions (r = 0.93±0.03, r = 0.89±0.03). Measured pressures correlated strongly (r = 0.98±0.02) when the device was placed in the bladder lumen. The survival animal initially recorded physiologic data, but later this deteriorated. However, endstage intraluminal device recordings correlated (r = 0.85±0.13) with the pressure catheter. Significant erosion of the implant through the detrusor was found.

**Conclusions:**

This study confirms correlation between suburothelial pressure readings and intravesical bladder pressures. Due to device erosion during ambulatory studies, a wireless implant is recommended for clinical rehabilitation applications.

## Introduction

Bladder management in patients with neurogenic bladder due to spinal cord injury (SCI) is necessary to avoid upper tract damage and incontinence. Upper tract damage, associated with high storage detrusor pressures and infections, was previously the leading cause of death in patients with SCI. However, with advances in urologic management, this is no longer the case [1]. Continued improvements in urologic rehabilitation and care may further improve life expectancy and quality of life for those with SCI. Pressure ulcers are a costly and potentially dangerous condition related to SCI, particularly when infection results from chronic moisture and pH changes due to urine leakage [2]. Additionally, decreased bladder control has been found to be one of the top five secondary conditions of SCI that patients find most distressing [3; 4]. Optimum management of neurogenic bladder would improve continence and decrease the risk of chronic moisture and potential secondary infections and septicemia.

Neuromodulation is one rehabilitation avenue for improved continence, as it has been shown to inhibit detrusor overactivity (DO) and promote urinary continence while maintaining low storage pressures [5–9]. The current method of continuous stimulation, however, may have several drawbacks that include neural habituation [5;10;11], tissue damage [5;12], and decreased battery life due to greater power dissipation. Event-based or conditional stimulation could minimize the limitations of continuous stimulation [9]. Conditional stimulation has the additional benefits of substantially decreasing stimulation time [12;13], extending electrode life [5;12], and preventing neural habituation [5;12].

Essential to the closed-loop operation of conditional stimulation is a safe and reliable method to monitor bladder activity and detect the onset of nascent hyperreflexive contractions [11;12;14;15]. Electrophysiological and patient-controlled methods to detect DO have been investigated in animals [16] and humans [12;14;15] but have not produced a clinically viable option to serve as a trigger [15]. Measurement of the accompanying rise in detrusor pressure has been a reliable technique in the laboratory [7;10;12;17]; however, due to infection, urethra trauma, stone-formation risks, and inconvenience, catheter-based systems do not provide a feasible means for chronic intravesical pressure monitoring [11;15]. An ideal chronic pressure sensor must be wireless, fully internalized, non-irritative, out of contact with the urine, and placed in a minimally-invasive manner. Triggered neuromodulation does not specifically require a highly accurate sensor, but does require rapid transmission of information on changes in bladder pressure that is highly time-correlated with contraction onset [18].

The long term goal of our research is to develop a small, wireless, catheter-free pressure monitor that meets these requirements and contains a wirelessly rechargeable battery and wireless data telemetry for cystoscopic implantation in the bladder in a suburothelial location. We hypothesize that implantation behind the urothelium will permit chronic retention of the device without impacting bladder health, that measured suburothelial pressure will correlate with intravesical pressure, and that bladder contractions may be identified without an abdominal reference catheter. The suburothelial, single-channel sensing modality, therefore, sacrifices absolute accuracy of pressure measurement with the understanding that triggered neuromodulation may be provided without high-precision pressure data [18].

**Fig 1:**
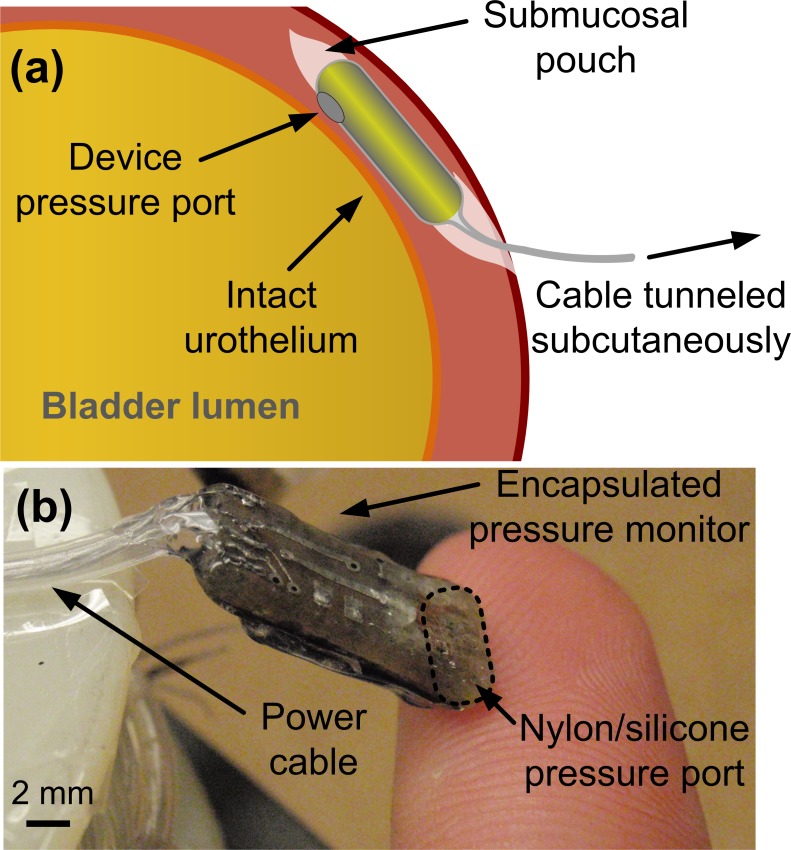
**(a)** Illustration of the catheter-free, wired pressure monitor as implanted within the bladder wall and **(b)** photograph of the fabricated implantable prototype.

In this new sensing modality there is a chance that localized detrusor contractions, changes in abdominal pressure, or rapid motion could create pressure artifacts superimposed on measured intravesical pressure, and so this study aims to evaluate the *in vivo* accuracy of our catheter-free pressure sensor [19;20] in the suburothelial location in several animals, using a wired pressure monitor prototype (Fig 1). We chose to use a wired prototype in these studies to focus on device validation, and to investigate the potentially deleterious effects of tethering the detrusor with a wired, submucosal implant.

## Materials and Methods

### Prototype Development

The prototype pressure monitor was constructed as previously reported [19]. In brief, the device consisted of a custom integrated circuit, a pressure sensor, and passive electronic components [19]. It was coated with silicone, a biocompatible packaging method suitable for chronically-implantable sensors, taking care to create a thin but durable silicone membrane over the pressure sensor diaphragm (Fig 1B). The submucosal portion of the prototype device measured 7.0 x 3.5 x 15 mm. A 4-lead, 100-cm long power/data cable constructed of stainless-steel leads inside a silicone tube was coupled to the device, and sealed with silicone to prevent moisture ingress.

The sensor received a clock input and power through the connected leads, and transmitted data at a rate of 4.9 kilo-samples/second. The sensor transmitted wireless data at the same rate, but only the level of received signal power was measured to confirm feasibility of wireless transmission at the implantation depth [19]. Digital data packets transmitted over both the wireless radio and the wired link were encoded with a specific signature so that reception errors (e.g. caused by wire damage, moisture ingress, etc) could be identified in real time. Bladder pressure data was captured digitally over the wired link and a custom receiver demodulated the data and reconstructed it for collection with a data acquisition system. The receiver maintained synchronization to the digital data stream and indicated when synchronization was lost, e.g. due to wire disconnect.

### Prototype Sensor Bench Test and Calibration

Prior to *in-vivo* studies, the prototype device pressure response was characterized through bench-testing using a small pressure chamber with a single lumen pressure sensor catheter for reference (SPR-524 Mikro-Tip Pressure Catheter, Millar Instruments, Houston, Texas). Measured sensor parameters previously reported [19;20] are omitted here for brevity, but are summarized in Table 1. The pressure sensitivity and function of prototype devices were calibrated just prior to implantation. To maintain device sterility, sensors were briefly tested at atmospheric pressure and after submerging in a 30-cm sterile water column in the operating room. The prototype device output sensitivity from this two-point calibration was assumed as constant for the duration of the corresponding animal study.

**Table 1:** Prototype Pressure Sensor Bench Test Summary.

Implantable device size	7.0 x 3.5 x 15 mm
Equivalent device French diameter	24 Fr
Sample rate	4900 samples/second
Sensing range	2200 cm H_2_O
Pressure sensitivity	0.8 cm H_2_O
Sensing accuracy (noise limited)	±1.6 cm H_2_O
Frequency response^†^	> 15 Hz

^†^Test limited by resonance frequency of pressure calibration chamber

### Animal Preparation

All animal care and experimental procedures were performed according to NIH guidelines and were reviewed and approved by the Institutional Animal Care and Use Committee of Case Western Reserve University and the Louis Stokes Cleveland VA Medical Center. Animals were purpose-bred for research use and procured from certified vendors. Experimental procedures were designed to minimize the potential for animal suffering, and were performed with veterinarian oversight. Furthermore, arterial pressure monitoring was used as a primary variable in monitoring anesthesia level under alpha-chloralose, to provide a more accurate measure of anesthesia depth.

A total of 3 in vivo experiments were performed: a nonsurvival feline, a nonsurvival canine, and an ambulatory survival canine. In all implant surgeries, animals were anesthetized using isoflurane (2% inhaled to effect) for surgical procedures and switched to alpha-chloralose (65 mg/kg) intravenously (IV) for urodynamics and device recording. Animals were given maintenance doses of alpha-chloralose IV as needed during the experiment. Vital signs were continuously monitored by anesthesia equipment, and were checked a minimum of hourly during anesthetized procedures. At the conclusion of terminal procedures, animals were euthanized with intravenous Sodium Pentobarbital (Euthasol, 5 mg/kg IV).

In all experiments the devices were implanted in an identical manner. First, the animal was placed supine and a midline abdominal incision was made. Dissection was performed down to the bladder. Perivesical fat was removed and a 1–2 cm incision was made in the detrusor, superficial to the urothelium. A pouch was created between the detrusor and the urothelium. The device was placed within the pouch with the gel pressure window facing the bladder lumen. The detrusor was then closed without tension using nonabsorbable suture.

### Non-Survival Studies

In the nonsurvival animals, a lumbosacral laminectomy (L6 –S2) was additionally performed and extradural cuff electrodes were implanted bilaterally on the S2 nerve roots. This procedure allowed for generation of on-demand bladder contractions in a superphysiological range to test the maximal sensing range of the implanted device. A multichannel, isolated voltage-controlled stimulator was used to generate stimulation waveforms. Detrusor contractions were evoked with constant-current bilateral S2 nerve root stimulation (20 Hz, 100 ms pulse width, balanced biphasic, first phase cathodic). Stimulation current was adjusted following a recruitment curve to generate peak bladder pressures greater than 80 cm H_2_O; approximately 1 mA stimulation amplitude was sufficient in canine and feline animals. Isometric physiologic contractions due to distension were also evoked in the nonsurvival animals with the urethra manually compressed.

### Urodynamics

With the animal anesthetized, a single lumen latex catheter was placed transurethrally to fill the bladder with saline from an external peristaltic pump. A reference pressure sensor catheter (SPR-524 Mikro-Tip Pressure Catheter, Millar Instruments, Houston, Texas) was also placed in the bladder transurethrally, adjacent to the filling catheter. The reference intravesical pressure signal was amplified and calibrated for hydrostatic offset (MPVS 300, Millar Instruments). Data from the reference and implanted device were recorded at 100 kHz using a digital acquisition system (PowerLab 8/30, AD Instruments, Colorado Springs, CO). Pressures were simultaneously transduced by the device and reference pressure sensing catheter placed in the bladder lumen. Both signals were digitally lowpass filtered to 0–10 Hz bandwidth after recording.

Bladder contractions were elicited both reflexively and electrically while data were recorded. The sensor was then explanted and the submucosal pouch was extended into the bladder lumen. Next the sensor was placed directly in the lumen, and the bladder was sutured closed. The bladder was then returned to the abdominal cavity. Simultaneous signals were again acquired with both sensors directly recording lumen pressure to investigate potential differences in sensor accuracy caused by the submucosal implantation location. At the end of the experiments the bladder was opened and the implant location was visually inspected from the lumen side to confirm mucosal integrity.

### Survival Ambulatory Study

In the survival animal, after implantation the device wires were passed through the abdominal wall. A trocar was used to subcutaneously tunnel the device leads to an exit site at the shoulder and the abdomen was closed. After survival surgery, the animal was continuously monitored until sternal recumbency was maintained (approximately 15–60 minutes). The animal was monitored twice daily postoperative and buprenorphine (0.2 mg/kg subcutaneous) and rimadyl (1mg/lb oral) were each given twice daily for a minimum of 2 days postop as analgesics. Additional doses were given if indicated after that.

After a 3-day healing period, ambulatory pressure recordings were collected every other day over 10 days of experimentation (n = 5 recording sessions). Testing was performed over 3 hours, twice daily. Device leads were connected to mobile instrumentation, which could follow the animal during ambulation. Recordings were performed while the animal was conscious and not confined to a cage to permit free ambulation and urination. The animal’s behavior was closely monitored and controlled to prevent damage and tugging to the implanted device cable. On post-operative day 13 the animal was anesthetized and urodynamics was performed to evaluate device recordings simultaneous with lumen reference catheter pressure recording as in the nonsurvival animals. The implant site was inspected to assess the bladder healing response and device condition. The implant was then removed from the suburothelial pouch, a transverse detrusor incision was made, the implant was placed directly in the bladder lumen, and the bladder was closed. Urodynamics was repeated to investigate differences in pressure recordings between implant and luminal positions. Finally, the animal was euthanized and necropsy was performed to visually assess the acute effects of device implantation. Specifically, the urothelium and detrusor around the device were visually examined to determine the tissue integrity and early signs of tissue erosion (degradation of tissue in response to device implantation) or device migration (movement of the device from the initial implant site caused by tissue erosion).

### Data Analysis

Obtained data were first segmented using LabChart 7 software. This step removed sections of data which were invalid due to experimental error, wiring issues, and/or loss of digital synchronization between the wired pressure monitor and external data receiver hardware. Corrupted data were visually determined to contain non-physiologic discontinuities typical of digital data errors. Data segments ranging from 0.5–16 minutes were obtained and analyzed.

These data segments were analyzed using MATLAB software. Data recordings were first lowpass filtered to a 0–10 Hz bandwidth and normalized to [0,1] by dividing reference pressure and prototype device readings by the full-scale range. Although implanted devices were calibrated prior to implantation, the data was normalized to permit comparison between different animals (feline vs. canine) and implant healing periods (anesthetized vs. survival). This also eliminated variable sensitivity between the different experiments caused by the implantation site (as discussed further on). Because the focus of this device was contraction detection for triggered neuromodulation—not absolute pressure measurement accuracy—comparisons between normalized reference and device recordings allowed for a fair judgement of the suburothelial device’s utility for contraction detection. Because ambulatory device recordings were made without a reference catheter, no quantitative data analysis was performed on these measurements. Ambulatory recordings, however, were qualitatively judged based on observations of animal behavior made simultaneously with pressure measurements.

Next, data recordings were analyzed for correlation between device and reference measurements. Two correlation coefficients were computed: full-length, average correlation, and windowed correlation coefficients corresponding to 10-s periods of measured high-frequency pressure changes, e.g. the sharp pressure increase at contraction onset. This analysis methodology was adopted so that the average, long-term sensor correlation could be separately reported from shorter-term correlation. This ensures that windowed correlation coefficients are not artificially skewed during long periods of bladder filling, and to provide some measure of correlation variance between multiple contractions in one recording. The device response to very slow or low-amplitude contractions not identified by the analysis methodology would be included in long-term, average sensor correlation values. In the context of conditional neuromodulation, sensor correlation to detrusor pressure during hyper-reflexive contractions is the critical requirement. Thus, the locations of recording windows were chosen to represent “active” bladder periods in which sensor feedback would be used to activate neuromodulation.

Data selection windows were obtained by processing recordings per the following analysis methodology. Data recordings were differentiated with respect to time, and windows were centered on local maxima demonstrating average pressure change rates with magnitudes above 3 cm H_2_O/second. The window selection threshold was chosen to be twice the average variance across all data sets such that only statistically significant pressure changes were selected for analysis. A window length of 10 seconds was chosen so that at least 8 windows were obtained for each full-length data recording, without any selection window overlap. Pearson’s correlation coefficients between reference and prototype were computed for each selected data window in MATLAB, and pooled statistics for each animal were computed with Minitab. Windowed correlation coefficients are here reported as the mean correlation ± one standard deviation range. Statistical box plots were produced to depict the interquartile range of measured correlation coefficients with box whiskers representing the full range of measured data after excluding statistical outliers identified with Peirce’s criterion.

All identified data windows were manually compared to experimental notes and reference recordings, and all analysis windows corresponded with observations of bladder activity. Note that this analysis methodology allows for calculation of mean and variance across many serial contractions obtained during one long anesthetized procedure, which could depict changes in sensor correlation which may occur as the bladder changes shape between contractions.

## Results

Bladder contractions were successfully recorded in all the animals. The animals showed differing levels of pressure attenuation when measured suburothelially. When the pressure monitor was explanted and placed directly in the lumen, a return to pre-implantation sensitivity was observed in all cases. Our conclusion therefore was that urothelial damping of vesical pressure occurred in all animals, and different levels of damping were caused by physiological differences and differing depths of surgical implantation. To reduce the influence of the variable mucosal damping effects, all recorded data traces were normalized, segmented, and analyzed as described in Methods. Despite the damping effect, correlation between suburothelial pressure and lumen pressure during contractions was noted in all animals.

**Fig 2:**
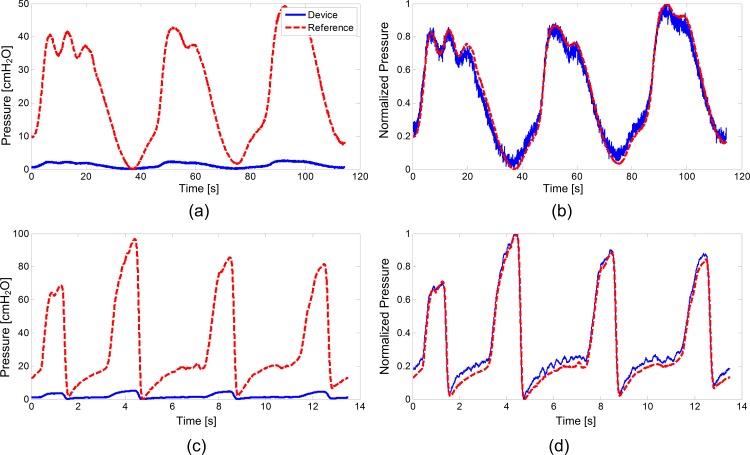
Feline Contraction Recordings. Examples of device (solid blue line) and reference (dashed red line) recordings of **(a)** bladder pressure as-recorded and **(b)** normalized contractions induced by electrical stimulation in the feline and **(c)** bladder pressure as-recorded and **(d)** normalized fast pressure changes due to manual bladder compression in the feline showing a faster rise time and slower decrease in pressures measured by the device.

**Fig 3:**
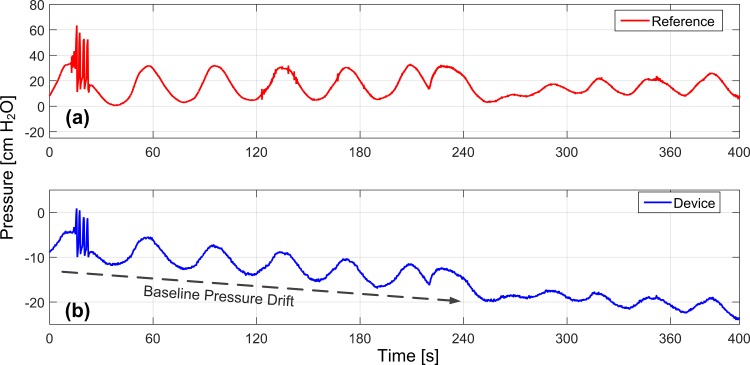
Suburothelial Baseline Drift. A slow drift of approximately 2 cm H_2_O per minute was noted between vesical reference **(a)** and suburothelial pressure monitor **(b)** recordings in the feline.

**Fig 4:**
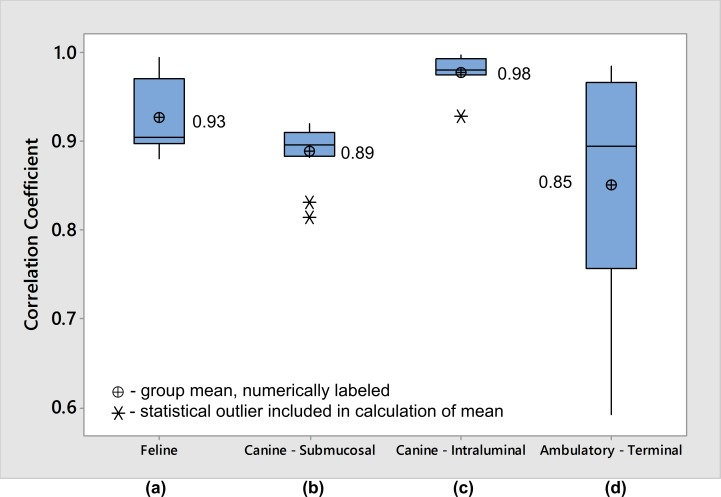
Pooled Multi-Contraction Correlation Coefficients from all animal experiments. Boxplots of measured correlation coefficients between prototype device and reference catheter in **(a)** anesthetized feline with submucosal device placement, **(b)** anesthetized canine with submucosal device placement, **(c)** anesthetized canine with intraluminal device placement, and **(d)** anesthetized canine terminal procedure with intraluminal device placement after 10-day implant period. Each box represents correlation coefficients obtained from 9–16 ten second recording windows comparing prototype device and reference catheter measurements. The box width depicts the interquartile range of measured correlation coefficients, while the box whiskers represent the full range of measured data after excluding statistical outliers identified with Peirce’s criterion. The central line within each box represents the median, and the data mean is indicated with a cross symbol. Raw data is shown in **S1 Table**.

In the nonsurvival feline experiment, phasic contractions were obtained by sacral nerve stimulation (Fig 2A and 2B). Rapid manual compression of the bladder produced elevated lumen pressures with an average one second length and peak pressures of 80–95 cmH_2_O (as measured by reference catheter) while the urethra was compressed to prevent leakage (Fig 2C and 2D). Prior bench-testing of implants confirmed a measurement accuracy of at least 2 cm H_2_O, and devices were calibrated for pressure sensitivity just prior to implantation. Calibrated pressure recordings from the device were attenuated compared to lumen pressure recordings from the reference pressure sensing catheter. After implantation, collected data also revealed a slow baseline drift (approximately 2 cm H_2_O per minute; Fig 3). However, after normalizing the data from the implanted device, and extracting 10-s recording windows around each contraction (n = 16) the windowed correlation between device and reference catheter data was similarly high during both evoked and manual compressions (r = 0.93±0.03; Fig 4). Average long-term correlation between the device and reference in this animal was also significant (r = 0.997).

**Fig 5:**
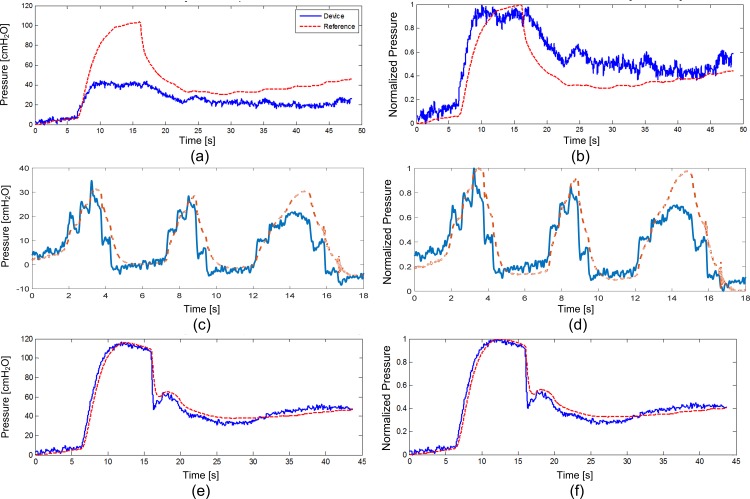
Example Recordings in the Nonsurvival Canine. Examples of device (solid blue line) and reference (dashed red line) recordings of electrically-induced isometric contractions in canine **(a)** as-recorded and **(b)** normalized recordings with the device in the submucosal location. Manual bladder compressions from the submucosal sensor **(c)** as-recorded and **(d)** normalized were correlated with reference lumen pressure but showed rate-change variation. Lumen pressure **(e)** as-recorded and **(f)** normalized recordings with the device in the bladder lumen showed good agreement with the reference.

In the nonsurvival canine experiment, bladder contractions evoked during sacral nerve stimulation were recorded and amplitude and rate-change variation between reference and implanted device was noted (Fig 5A and 5B). The discrepancy was repeatable over several contractions (n = 12), and this reduced the windowed correlation between device and reference (r = 0.89±0.03; Fig 4) and average long-term correlation coefficient (r = 0.893). Faster rise time was noted in the implanted device at the contraction onset, and slower pressure decrease after the peak (Fig 5A and 5B). Manual compressions of the bladder produced a similar rapid rise effect as that noted during contraction measurement (Fig 5C and 5D). After the device was explanted and placed in the lumen, the rate-change variation in measured pressure data vanished (Fig 5E and 5F). With the device in the lumen, windowed ten second recordings (n = 9) correlated strongly between the device and the pressure sensing catheter (r = 0.98±0.02; Fig 4). Long-term correlation in this mode of testing was high and roughly equivalent to the windowed correlation (r = 0.983).

During post-operative healing in the survival canine experiment, the animal scratched at the wired device interface cable, and damaged several conductors, causing intermittent short-circuiting. The implanted sensor used a low voltage power supply so there was no trauma or risk to the animal. However, due to the unreliable cable integrity, significant periods of invalid data were recorded. Because the implant transmitted data samples as digital packets, the external receiver synchronized to the digital pattern of received data and valid data could be recognized. Loss of synchronization was noted and used to segment the data in post-experiment analysis. Other digital reception errors manifested as discontinuous jumps in pressure data, which provided clear indication for data segmenting. After segmentation, each ambulatory recording session produced several analyzable data segments, which were analyzed in relation to observations of animal behavior.

**Fig 6:**
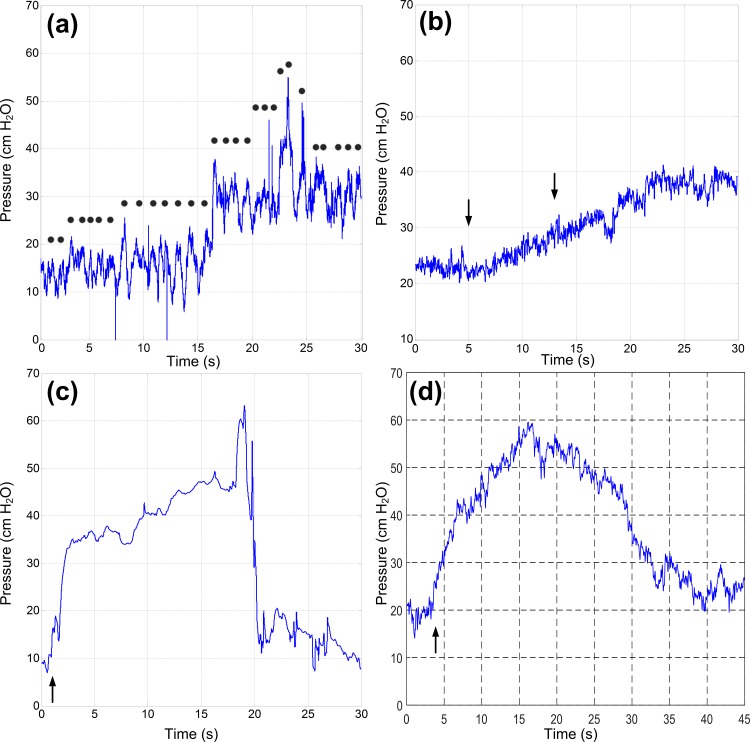
Examples of Pressure Data from the Device during Ambulation. Data captured in the ambulatory canine due to **(a)** walking (step frequency indicated approximately by dots) and **(b)** sit-to-stand posture change (black arrows indicate approximate start/stop of motion). Bladder contractions (onset denoted by black arrow) measured in ambulatory canine showing both **(c)** voiding and **(d)** non-voiding contractions.

Early ambulatory pressure recordings in the survival implanted canine were consistently within physiologic range. Presumed motion artifacts were observed by noting animal movement without apparent urination or squatting behavior. Pressure data collected during these periods were attributed to abdominal pressure changes from changes in position and other motions superimposed on vesical pressure; however, validation was not possible since confirmatory catheter-based pressure recordings were not made during ambulation. Nonetheless, pressure data during motion had high-frequency elements (Fig 6A) corresponding with periodic animal motion, enabling qualitative validation. Slow posture changes showed gradual shifts in pressure baseline over tens of seconds occurring after completion of visible movement (Fig 6B), potentially caused by physiologic changes in abdominal pressure. Voiding and non-voiding bladder contractions were confirmed by observing animal behavior; detrusor trauma from the implant caused frequent bladder spasms in which the animal would crouch and attempt to urinate. Corresponding device recordings during these periods generally matched physiologic pressure signal shapes (Fig 6C and 6D). These non-voiding contractions occurred over a long period.

More variable recordings in a non-physiologic range were found in later ambulatory recordings, indicating migration of the device from the bladder wall. During the anesthetized terminal procedure, the implanted device did not produce correlated pressure data from the original implant location. After the device was explanted from the submucosa and placed directly in the lumen, the sensor correlated well (r = 0.85±0.13; Fig 4) with the reference catheter during manual bladder compressions (n = 10).

During necropsy, the condition of the implant was assessed by visually analyzing the bladder from both mucosal and detrusor sides. Despite the short implant duration, significant damage to the implanted device and the detrusor was apparent. The sutures securing the device within the submucosal pouch were present, but the detrusor around the suture had eroded. Consequently, the sutures were no longer tight and about half of the submucosal portion of the device protruded through the detrusor. Furthermore, damage to the device power/data cable had occurred where the cable passed through the sutured tissue. Damage to the cable caused intermittent open-circuit failures in two of the four conductors within the cable.

## Discussion

While urodynamics, uroflow, and pressure-sensing catheters give physicians insight into the overall health of a patient’s bladder, they are limited in chronic utility for contraction detection and neuromodulator control. Implantable sensors offer the opportunity for chronic monitoring to confirm treatment efficacy or enable conditional neuromodulation. Previously published descriptions of implantable bladder sensors have required an open approach, percutaneous wires, or both [21–23]. Unlike existing pressure-sensing implants [21;24–29], this pressure monitor was specifically developed for cystoscopic implantation and wireless catheter-free chronic application in the bladder. In this early feasibility study, however, a simpler, wired version of the pressure monitor was tested to investigate the fidelity of suburothelial contraction detection and feasibility of chronic retention of a wired device within the bladder wall. Confirmation of the value of suburothelial pressure monitoring is required prior to further development of this sensing modality.

This study is limited in its small sample size and short duration, and contains no replications in each animal. We therefore cannot conclusively comment on the reliability of using a submucosal pressure sensor to infer detrusor pressures. Furthermore, without a reference catheter placed during ambulatory recordings, we can only hypothesize that recorded pressure changes occurring without urination or squatting behavior were attributed to abdominal pressure changes. It is possible that other biomechanical forces acting on the implanted sensor could have caused changes in measured pressure during ambulation.

Our initial findings, however, show that pressures measured submucosally are well correlated with lumen pressures. The correlation coefficient is high enough to detect bladder events such as detrusor contractions or abdominal compressions.

One caveat to our findings is that the biomechanics of the implant site–where the pressure monitor measures a combination of hydrostatic lumen pressure and localized, directional force from the detrusor muscle–limits pressure measurement accuracy. We observed two pressure measurement abnormalities in this study. First, during bladder contractions, the implanted device showed a faster rise time at contraction onset and slower pressure decrease after contraction peak, compared to an intralumenal reference. This effect was likely due to the force of the detrusor pressing on the back of the implant, as this finding resolved with placement of the sensor in the bladder lumen. Although we only observed this effect in two nonsurvival animal studies and therefore cannot make concrete conclusions, we believe this may be a potential advantage of submucosal pressure recording. If detrusor contraction onset could be reliably detected prior to a corresponding rise in vesical pressure, it could enable faster feedback to a stimulator for conditional or closed-loop stimulation.

In addition, the device and reference pressure recordings were not identical in range, despite showing high correlation. Baseline pressure offset drift was only noted in the non-survival feline, but reduced sensitivity was observed in all animals. This drift and attenuation was not observed in bench calibration of the device, and may be due to a physiologic effect of suburothelial placement. It is possible that the urothelium provides a “damping” effect which reduces pressure sensitivity, and entrapped urothelial air bubbles could explain the slow drift noted in one animal.

The device can be calibrated against lumen pressure at the moment of implantation to increase immediate accuracy, but biological changes or fibrosis around the implant site could necessitate regular maintenance calibrations against a catheter reference. These changes would complicate calibration and long-term accuracy of the implanted device, but practical suburothelial contraction detection may still be feasible despite these effects. This study was not designed to measure these effects, as simple correlation between device and reference was the desired response variable. While these effects limited the accuracy of the pressure monitor, we had no issues observing bladder contraction onset, critical for the application of triggered neuromodulation.

A clear outcome with the wired pressure monitor in the survival animal was a loss of reliable recordings after several days of implantation. This was likely caused by erosion of the sensor through the detrusor and damage to the device cables. The observed damage to both detrusor and device was likely caused by repeated cyclic tensioning of the device cable, which loosened the detrusor suture and allowed the sutures to partially saw through the cable. While we only studied this effect in a single ambulatory animal, the rapid loss of device integrity and observed detrusor trauma highlights the need for a wireless device to limit bladder tethering, erosion, migration, and damage to the device. Although further studies with wireless devices are clearly needed, this study is significant in that it demonstrates the feasibility of suburothelial ambulatory pressure monitoring.

## Conclusions

In conclusion, we have demonstrated that bladder pressure measured suburothelially correlates positively with intravesical pressure. Despite a damping effect when measuring pressure from this location, our data suggest that bladder contractions may be reliably observed from a single-channel submucosal pressure sensor. This observation motivates continued development of transurethrally implantable sensors for rehabilitation of neurogenic bladder, e.g. via conditional neuromodulation. In addition, erosion in the acute survival experiment likely due to tethering of wires in this prototype indicates the necessity for further development of a wireless sensor. Such a device would enable future studies focusing on the comparative accuracy of suburothelial bladder pressure measurement.

## Supporting Information

S1 Table Correlation coefficients extracted from 10‐s windows of raw data. These data are plotted as boxplots in Fig 4.
(PDF)

## Abbreviations

SCI: Spinal cord injured/injury
DO: Detrusor overactivity
cm H_2_O: Centimeters of water (unit of pressure)
