# Supplementary material for: Suburothelial Bladder Contraction Detection with Implanted Pressure Sensor
Source: PLoS One. 2017 Jan 6;12(1):e0168375. doi: 10.1371/journal.pone.0168375 (PMC5218553; doi:10.1371/journal.pone.0168375)
Supplement: S1 Table — These data are plotted as boxplots in Fig 4. (PDF) [file pone.0168375.s001.pdf]

Supplementary Information for: Suburothelial Bladder Contraction Detection with Implanted Pressure Sensor

Correlation coefficients extracted from 10-s windows of raw data
These data are plotted as boxplots in Fig. 4

Feline	Canine - Submucosal	Canine - Intraluminal	Ambulatory - Terminal
0.897	0.911	0.990	0.708
0.892	0.891	0.929	0.774
0.981	0.815	0.995	0.937
0.904	0.920	0.980	0.983
0.928	0.900	0.977	0.881
0.974	0.907	0.974	0.907
0.906	0.915	0.997	0.777
0.952	0.832	0.978	0.962
0.994	0.889	0.981	0.593
0.881	0.891		0.984
0.899	0.882		
0.897	0.908		
0.890			
0.904			
0.976			
0.963			
